# Baseline Lower Urinary Tract Symptoms and Sexual Dysfunction in Men with Newly Diagnosed Localized Prostate Cancer

**DOI:** 10.3390/jcm12134462

**Published:** 2023-07-03

**Authors:** Wan Song, Jun-Seop Kim, Kwang Jin Ko

**Affiliations:** Department of Urology, Samsung Medical Center, Sungkyunkwan University School of Medicine, Seoul 06351, Republic of Korea; wan.song@samsung.com (W.S.); junseop92.kim@samsung.com (J.-S.K.)

**Keywords:** lower urinary tract symptoms, prostate cancer, sexual dysfunction

## Abstract

We evaluated baseline lower urinary tract symptoms (LUTS) and sexual dysfunction in patients with newly diagnosed localized prostate cancer. Data were obtained from a cohort registry of patients with localized prostate cancer scheduled for radical prostatectomy. Before surgery, patients completed a 3-day voiding diary; International Prostate Symptom Score (IPSS), International Index of Erectile Function-5 (IIEF-5), and Expanded Prostate Cancer Index Composite (EPIC) questionnaires; and a urodynamic study. Data were analyzed according to benign prostatic hyperplasia treatment status and age group. In total, 380 patients (median age, 67 years) were enrolled in this study. On the IPSS, 10.8% of patients had severe symptoms. On the IIEF-5, 8.7% of patients did not have erectile dysfunction and 52.9% had moderate-to-severe erectile dysfunction. On the EPIC, 3% of patients indicated that they did not have urinary control and only 13% responded that their erectile function was good or very good. The mean IPSS and IIEF-5 scores showed significant differences among age groups. Thus, patients with localized prostate cancer show various LUTS and sexual dysfunction at baseline, and these symptoms worsened with increased age.

## 1. Introduction

The prevalence of prostate cancer is rapidly increasing in Asian countries, including South Korea, due to Westernized diets, increased screening for prostate cancer, and rapid aging of the population. In particular, prostate cancer is expected to become more frequent in men aged ≥65 years [1]. On the other hand, the 5-year survival rate is currently high (94.4%) and is as high as 98.6% for localized prostate cancer [2]. As the proportion of surviving patients increases owing to this high survival rate, interest in the impact of prostate cancer on the quality of life (QoL) of survivors has increased. Although most studies to date have focused on the causes and prevention of common postoperative complications such as urinary incontinence and erectile dysfunction (ED) [3,4,5], patients diagnosed with prostate cancer are typically middle-aged or older men at risk of lower urinary tract symptoms (LUTS).

Many patients who receive treatment for LUTS caused by benign prostate hyperplasia (BPH) are subsequently diagnosed with prostate cancer during follow-up. Moreover, one study found that approximately 45% of patients with newly diagnosed prostate cancer had moderate-to-severe LUTS [6]. Additionally, a large-scale epidemiological study conducted in South Korea found that approximately 45% of patients aged >40 years complained of moderate-to-severe LUTS based on survey data [7]. Thus, it can be estimated that approximately 40% of men >40 years of age have LUTS, regardless of the presence of prostate cancer. However, the prevalence of overactive bladder at the time of prostate cancer diagnosis varies across studies. For example, in a survey-based study, the proportion of patients with prostate cancer with overactive bladder symptoms was as high as 55.8% [6]. Another study found that approximately 11–61% of patients have detrusor overactivity confirmed by urodynamic studies before radical prostatectomy [8].

The increase in life expectancy after radical prostatectomy should be examined in terms of the rates of LUTS and sexual function. However, few studies have rigorously and prospectively evaluated urinary symptoms or QoL in patients with prostate cancer using tools such as voiding diaries, disease-specific questionnaires, or validated health-related QoL questionnaires. A clear evaluation of LUTS and sexual dysfunction at baseline is important in determining treatment strategies according to symptomatic changes after prostate cancer treatment. Therefore, this study aimed to identify current rates of LUTS and sexual dysfunction in patients newly diagnosed with prostate cancer using prospective registry data.

## 2. Materials and Methods

### 2.1. Patients

In this study, baseline LUTS, sexual function, and health-related quality of life were assessed in a registry established for one year from July 2022 in patients diagnosed with localized prostate cancer (T2-T3a N0 M0) and scheduled for radical prostatectomy. Exclusion criteria included those diagnosed with urinary malignancy, such as bladder cancer; those who had received prior chemotherapy or pelvic radiation therapy before prostatectomy; and those who had undergone urethral surgery. This study was approved by our institutional review board (IRB No. 2021-04-159).

### 2.2. Assessment

We assessed the baseline status of patients diagnosed with localized prostate cancer using several questionnaires and a 3-day voiding diary. The baseline survey was performed on the day of the diagnosis of localized prostate cancer, before radical prostatectomy. Participants completed the International Prostate Symptom Score (IPSS) questionnaire, a widely used tool for evaluating symptoms related to male LUTS/BPH. The IPSS consists of seven items, with higher scores indicating more severe symptoms. Additionally, participants completed the Expanded Prostate Cancer Index Composite (EPIC) questionnaire [9], which evaluates health-related QoL in patients with prostate cancer in terms of urinary, bowel, sexual, and hormonal domains. Each domain is divided into functional and bother subscales. In addition, the urinary domain is subdivided into incontinence and irritative/obstructive subscales. The EPIC questionnaire consists of 50 items, with higher scores indicating better QoL. Participants also completed the International Index of Erectile Function-5 (IIEF-5) questionnaire, which is a tool for evaluating male sexual function. The IIEF-5 consists of five items, with higher scores indicating better sexual function. Finally, we collected data on patients’ urinary habits and patterns using a 3-day voiding diary, which involved recording the frequency of voiding and any episodes of incontinence or urgency, as well as the voided urine volume.

### 2.3. Urodynamic Study

Before cystometry, the maximum flow rate (Qmax) was measured using simple uroflowmetry, and the post-voided residual urine volume (PVR) was measured. During filling cystometry, bladder sense and cystometric capacity were measured. Detrusor overactivity was documented when an involuntary detrusor contraction (IDC) was identified. During voiding cystometry, detrusor pressure at the maximum flow rate (PdetQmax) was measured, and the bladder outlet obstruction index (BOOI, PdetQmax − 2Qmax) and bladder contractility index (BCI, PdetQmax + 5Qmax) were calculated from the results.

### 2.4. Statistical Analysis

Descriptive statistics for continuous and categorical variables are presented as the mean (standard deviation) and frequency (%), respectively. Patients were stratified into three age groups: <65 years, 65–75 years, and ≥75 years. The mean and standard deviation of each group were calculated for all variables. One-way analysis of variance (ANOVA) was performed to compare the three groups, followed by post hoc analysis using the Bonferroni test.

Statistical analyses were performed using SPSS version 27 (IBM Corporation, Armonk, NY, USA). All inferences and descriptive *p*-values are based on two-tailed tests. Statistical significance was set at *p* < 0.05.

## 3. Results

### 3.1. Baseline Characteristics

A total of 380 patients diagnosed with localized prostate cancer were enrolled in preoperative baseline investigations. The median age was 67 years, 49.7% of patients were diagnosed with BPH and were taking BPH medication, and 2.4% of patients underwent BPH surgery. In total, 12.9% of patients were currently taking anticholinergics and mirabegron for overactive bladder symptoms. According to 3-day voiding diary data, the mean number of daytime frequency and nocturia episodes per day was 6.6 and 1.1, respectively. The mean number of urgency and urgency urinary incontinence episodes per day was 1.3 and 0.04, respectively. The mean estimated prostate size was 34.7 gm. On the IPSS, 10.8% of patients complained of severe symptoms. On the IIEF-5, 8.7% of patients did not have ED and 52.9% had moderate-to-severe ED. On the EPIC, the mean sexual summary score was very low, at 40.5, whereas the mean urinary, bowel, and hormonal summary scores were high, at 85.1 to 92.8. Table 1 summarizes the baseline characteristics of the study population.

### 3.2. Characteristic EPIC Item Results

**Figure 1:**
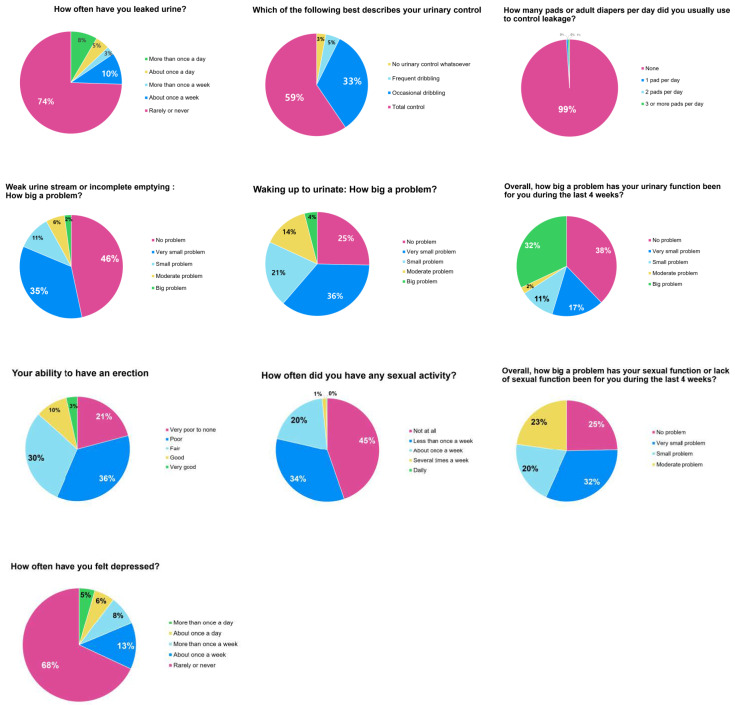
Characteristics of EPIC items.

Figure 1 presents the results of relevant EPIC questionnaire items. Regarding the frequency of urine leakage, 74% indicated that such episodes occurred rarely or never and 8% indicated that they occurred more than once per day. Additionally, 99% of patients indicated that they did not use pads or adult diapers, while 1% indicated that they used more than three sheets per day. Regarding urinary control, 59% indicated that they had total control, 33% indicated occasional dribbling, and 3% indicated that they did not have urinary control. Regarding a weak urine stream or incomplete emptying, 46% indicated that this was not a problem, 35% indicated that it was a very small problem, and only 8% indicated that it was a moderate-to-big problem. Regarding nocturia, 25% of patients indicated that it was not a problem and 36% indicated that it was a very small problem, while 18% indicated that it was a big problem.

At baseline, only 13% of patients indicated that their erectile function was good or very good, and 57% indicated that it was poor to none. In total, 45% of patients indicated that they had not engaged in any sexual activity in the previous four weeks, 34% less than once a week, and 20% about once a week. Regarding overall sexual function or lack of sexual function, 25% of patients indicated that there was no problem and 32% of patients indicated that there was a very small problem.

Regarding depression, 68% of patients indicated that they rarely or never felt depressed, while 11% of patients indicated that they felt depressed about once or more than once a day.

### 3.3. LUTS and Sexual Funciton According to Current BPH Medication

Table 2 presents the results according to current BPH treatment. Patients taking BPH medication (alpha blocker only) were older and had a larger prostate size than those not taking BPH medication, but there was no difference in prostate-specific antigen levels. Qmax and PVR did not differ between the two groups. Daytime frequency and urgency frequency were significantly higher and mean bladder capacity was lower in patients taking BPH medication than in those not taking BPH medication. Further, the IIEF-5 total score and IPSS total score, voiding and storage subscores, and QoL were all significantly worse in patients taking BPH medication than in patients not taking BPH medication. On the EPIC, only the urinary and sexual summary scores were significantly lower in patients taking BPH medication than in those not taking BPH medication.

### 3.4. LUTS and Sexual Function According to Age Group

There were no significant differences among age groups in the mean number of daytime voids in the 3-day voiding diary. However, the nocturnal polyuria index (*p* < 0.001) and the mean number of nocturia episodes per day (*p* < 0.001) increased significantly with increased age. Additionally, as age increased, the mean number of urgency episodes per day tended to increase (*p* = 0.052) (Table 3).

A significant decrease in Qmax was observed with increased age. Residual urine volume also significantly increased with increased age. The proportion of patients with IDC tended to increase with increased age (from 25.5% in those aged <65 years to 33.2% in those aged 65–75 years and 41.7% in those aged ≥75 years); however, the differences did not reach significance. Regarding bladder sensation during filling cystometry, the first sensation of bladder filling, first desire to void, and strong desire to void volumes tended to decrease with increased age, but the differences did not reach significance. Maximal cystometric capacity was significantly smaller in patients aged ≥75 years (326.9 cc) than in those aged >65 years (371.9 cc) (*p* = 0.001). The incidence of bladder outlet obstruction significantly worsened with increased age (*p* = 0.031) and the proportion of patients with weak bladder contractility increased significantly with increased age (*p* = 0.035) (Table 3).

**Figure 2:**
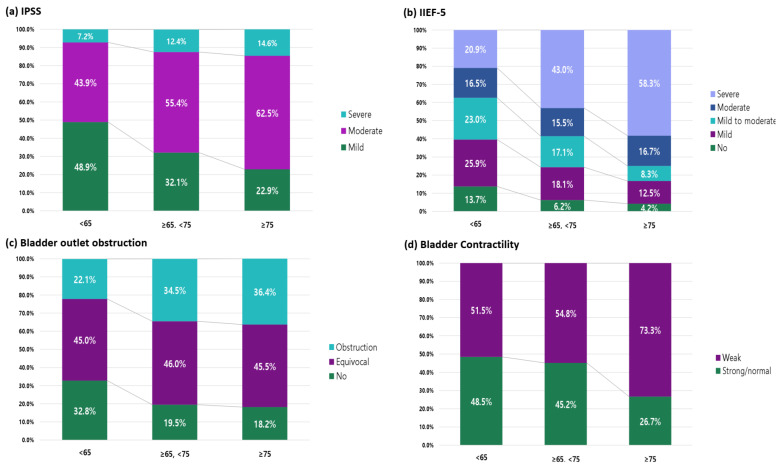
Proportions are shown according to age group for (**a**) the mean International Prostate Symptom Score (IPSS), (**b**) the mean International Index of Erectile Function-5 (IIEF-5) score, (**c**) bladder outlet obstruction, and (**d**) bladder contractility.

The mean IPSS showed significant differences among the three age groups for total, subscale, and QoL scores. However, in post hoc analysis, there was no difference in the IPSS voiding subscore among age groups. The IPSS total score, storage subscore, and QoL scores were significantly different between patients aged <65 years and those aged ≥75 years (Table 3). Regarding symptom severity, severe symptoms were reported in only 7.2% of patients aged <65 years and 14.6% of patients aged ≥75 years. Conversely, 48.9% of patients aged <65 years and 22.9% of patients aged ≥75 years reported mild symptoms (Figure 2).

The mean IIEF-5 total score was significantly different among age groups; in post hoc analysis, IIEF-5 scores were significantly decreased with increased age (Table 2). Among patients aged <65 years, 13.7% did not have ED; however, among patients aged ≥75 years, only 4.2% did not have ED, 16.7% complained of moderate ED, and 58.3% complained of severe ED (Figure 2).

In the health-related QoL domain summary scores on the EPIC questionnaire, urinary (*p* = 0.180), bowel (*p* = 0.773), and hormonal (*p* = 0.453) domain summary scores were not significantly different among age groups; however, the sexual domain summary score was significantly different among the age groups. The sexual domain score was low (46.4 ± 21.2) in those aged <65 years but was significantly lower in those aged 65–75 years (38.1 ± 18.5) and ≥75 years (33.4 ± 16.6) (*p* < 0.001). Urinary domain incontinence subscores were significantly lower in patients aged 65–75 years and ≥75 years than in those aged <65 years, while there were no differences among age groups in the other urinary domain subscales. Sexual domain function subscores were significantly lower with increased age, while there were no differences among age groups in the sexual domain bother subscores (Table 3).

## 4. Discussion

We comprehensively evaluated the baseline status of patients with localized prostate cancer prior to surgery and observed various types of LUTS and sexual dysfunction. Although several previous studies evaluated 3-day voiding diary and urodynamic study data at baseline, the present study adds to the literature because of its high voiding diary response rate and urodynamic study implementation rate in 380 patients. In addition, in the present study, all measure of LUTS and sexual dysfunction were investigated immediately after diagnosis and before radical prostatectomy; therefore, there was little recall or selection bias.

In a study on the prevalence of LUTS (based on the IPSS) in Asian men aged ≥40 years, mild symptoms were reported in 51.4%, moderate symptoms in 29.1%, and severe symptoms in 7.2% of patients [7]. Even considering the higher age in the present study than in this previous population-based study, the proportion of patients with localized prostate cancer already reporting moderate-to-severe symptoms was high at 62.9%. Age at the time of diagnosis may be assumed as the most important risk factor for LUTS. In particular, the mean number of nocturia episodes was greater in patients aged ≥75 years due to an increase in nocturnal urine volume along with a decrease in mean bladder capacity compared to younger age groups. In a study of men in their 60s–80s, the prevalence of nocturnal polyuria syndrome was reported to be 17–41% [10]. Thus, although nocturia does not occur because of prostate cancer, it is necessary to note that many patients with prostate cancer have nocturia given the age at which prostate cancer occurs. In the present study, with increased age, the maximum flow rate decreased significantly, the amount of residual urine increased, and the incidence of IDC tended to increase. The debate surrounding the effect of radical prostatectomy on the development of detrusor overactivity is contentious. Detrusor overactivity in 2–77% of patients appears to be a de novo dysfunction and persists in 83% of patients after radical prostatectomy [11,12]. In the present study, approximately 42% of patients aged ≥75 years had accompanying detrusor overactivity, and the prevalence of basal detrusor overactivity may increase as the age of surgery increases.

Changes in these indicators with increased age were reflected in the IPSS categories. Surprisingly, however, there were no significant differences according to age group in the function, bother, and irritative/obstructive subscales of the EPIC urinary subscale, and only the incontinence item showed a significant decline in QoL with increased age. Urinary incontinence after radical prostatectomy or radiation treatment is a frequent complication with a potentially devastating impact on QoL. However, an interesting result was that not all patients were free of urinary incontinence before surgery. Urinary control was not possible in 3% of patients, 8% of patients experienced urinary incontinence more than once a day, and even 1% of patients used three or more sheets per day. Aboseif et al. [13] noted that urodynamics is useful in assessing the risk of incontinence, particularly for asymptomatic patients prior to surgery. The postoperative incontinence rate was 3% when detrusor function was normal preoperatively but ranged between 17 and 71% in the presence of any preoperative abnormality. Therefore, in older adult patients aged ≥65 years, checking the baseline incontinence status with urodynamic studies before prostate cancer treatment is considered helpful for inferring and treating symptomatic changes after treatment.

In our study population, approximately half of the patients were still taking BPH medication. Among patients diagnosed with localized prostate cancer, those under and not under maintained BPH treatment showed a low mean maximum flow rate with no significant difference, and there was also no difference in the PVR. Further, in the urodynamic study, there was no difference in the degree of obstruction or contractility. These objective test results indicate no significant difference between patients receiving and not receiving BPH treatment, which is mainly related to voiding symptoms.

The voiding diary findings of the patients who did not take BPH medication were clinically insignificant. However, the mean numbers of daytime urinary voids, nocturia episodes, and urgency episodes in the 3-day voiding diary and symptoms on health-related QoL questionnaires were more severe in patients taking BPH medication than in those not taking BPH medication. It seems reasonable to assume that symptoms in patients initially diagnosed with localized prostate cancer are caused by prostate enlargement.

Sexual activity is common in older males and is an important part of the QoL of older men [14]. ED is a frequent complication of radical prostatectomy [15]. Although the symptoms vary in severity, approximately 91% of patients already have ED before radical treatment. When asked about the frequency of sexual intercourse, 45% indicated no sexual intercourse in the previous four weeks and 34% indicated a frequency of less than once a week. More patients than expected did not have a regular sexual life, which is very likely due to ED. Interestingly, many patients showed a significant decrease in sexual function with increased age, but there was no difference in the degree to which sexual function affected their QoL.

This study has several limitations. First, all patients were scheduled to undergo robot-assisted radical prostatectomy. In South Korea, robotic surgery is not eligible for national reimbursement, and patients who could afford this expensive surgery were included as eligible patients. Considering their socioeconomic status, it is possible that many patients who are actively interested in LUTS or sexual function problems are more likely to have access to medical care. Second, we did not analyze the association between LUTS and sexual dysfunction in patients with diagnosed prostate cancer. Although it is considered an established theory that BPH does not cause prostate cancer, no analysis of its symptomatic relationship has been performed. However, in the present study, the risk of recall bias was very low, as the questionnaire survey targeted patients waiting for surgery at the time of the diagnosis of prostate cancer. In addition, a great advantage of the present study is that it was designed with the intention of collecting voiding diary data and conducting urodynamic studies in all patients.

## 5. Conclusions

Patients with localized prostate cancer show various types of LUTS and sexual dysfunction before radical prostatectomy, and their symptoms are worse with increased age. Although radical treatment of localized prostate cancer is an important factor in patient survival, it is necessary to accurately understand the status of LUTS and sexual dysfunction before treatment, as this can affect QoL after treatment.

## Figures and Tables

**Table 1:** Baseline characteristics.

N	380
Age, median (range)	67 (35–83)
≥65 years	241 (63.4%)
≥75 years	48 (12.6%)
BMI	24.8 ± 2.7
Smoking	
None	27.9% (*n* = 106)
Ex-smoker	64.2% (*n* = 244)
Current smoker	7.9% (*n* = 30)
DM	20.0% (*n* = 76)
Previous BPH surgery	2.4% (*n* = 9)
Concurrent BPH medication	49.7% (*n* = 189)
Concurrent OAB medication	12.9% (*n* = 49)
PSA	11.3 ± 11.5
Transrectal US	
Total prostate size (gm)	34.7 ± 15.3
Transitional zone size (gm)	16.1 ± 11.4
Simple uroflowmetry	
Maximal flow rate	12.4 ± 5.5
Voided volume	238.4 ± 95.5
PVR	42.5± 48.4
3-day voiding diary	
Daytime frequency	6.6 ± 2.1
Nocturia	1.1 ± 0.8
Urgency	1.3 ± 2.4
Urgency urinary incontinence	0.04 ± 0.30
Mean bladder capacity	227.9 ± 76.4
Total urine volume	1672.1 ± 589.9
Nocturnal urine volume	466.6 ± 339.6
Nocturnal polyuria index	0.27 ± 0.17
IPSS total	10.6 ± 6.8
IPSS voiding subscale	5.8 ± 4.5
IPSS storage subscale	4.8 ± 3.1
IPSS QoL	2.5 ± 1.5
Mild	37.1%
Moderate	52.1%
Severe	10.8%
IIEF-5 total	10.6 ± 6.8
No ED	8.7%
Mild	20.3%
Mild to moderate	18.2%
Moderate	16.1%
Severe	36.8%
EPIC	
HRQOL domain summary score (0–100)	
Urinary summary	85.1 ± 13.2
Bowel summary	92.8 ± 8.4
Sexual summary	40.5 ± 19.8
Hormonal summary	89.8 ± 11.9
Domain-specific HRQOL subscale (0–100)	
Urinary subscales	
Function	91.0 ± 12.5
Bother	81.9 ± 14.6
Incontinence	89.8 ± 13.8
Irritative/obstructive	85.7 ± 12.2
Bowel subscales	
Function	91.7 ± 9.9
Bother	94.5 ± 9.1
Sexual subscales	
Function	33.0 ± 21.3
Bother	58.3 ± 29.3
Hormonal subscales	
Function	86.1 ± 14.9
Bother	93.2 ± 9.8

BPH, benign prostate hyperplasia; EPIC, Expanded Prostate Cancer Index Composite; HRQOL, health-related quality of life; IIEF-5, International Index of Erectile Function-5; IPSS, International Prostate Symptom Score; OAB, overactive bladder, PVR, post-voided residual urine volume; QoL, quality of life.

**Table 2:** Characteristics of patients taking or not taking concurrent BPH medications.

	BPH Medication	Non-BPH Medication	*p*
N	189	191	
Age	67.9 ± 6.3	65.1 ± 7.3	<0.001
PSA	10.7 ± 10.9	11.9 ± 12.1	0.285
Transrectal US			
Total prostate size (gm)	37.2 ± 17.1	32.2 ± 12.8	0.001
Transitional zone size (gm)	17.6 ± 12.7	14.6 ± 9.7	0.051
Simple uroflowmetry			
Maximal flow rate	12.0 ± 5.6	12.7 ± 5.3	0.182
PVR	43.7 ± 49.3	41.2 ± 47.6	0.621
Urodynamic study			
BOOI	33.4 ± 19.9	33.5 ± 18.4	0.949
BCI	101.6 ± 68.8	96.0 ± 31.0	0.313
3-day voiding diary			
Daytime frequency	7.0 ± 2.2	6.2 ± 1.8	<0.001
Nocturia	1.2 ± 0.9	0.9 ± 0.8	0.001
Urgency	1.6 ± 2.8	1.0 ± 1.9	0.015
Urgency urinary incontinence	0.03 ± 0.16	0.05 ± 0.41	0.448
Mean bladder capacity	215.0 ± 72.9	241.1 ± 77.7	0.001
Nocturnal polyuria index	0.29 ± 0.17	0.25 ± 0.17	0.023
IPSS total	12.4 ± 7.2	8.9 ± 5.8	<0.001
IPSS voiding subscale	6.8 ± 4.8	4.8 ± 4.0	<0.001
IPSS storage subscale	5.6 ± 3.2	4.1 ± 2.9	<0.001
IPSS QoL	2.9 ± 1.5	2.1 ± 1.5	<0.001
IIEF-5 total	10.5 ± 5.9	13.5 ± 6.3	<0.001
EPIC			
HRQOL domain summary score			
Urinary summary	82.6 ± 13.3	87.5 ± 12.6	<0.001
Bowel summary	92.2 ± 9.4	93.3 ± 7.4	0.212
Sexual summary	35.8 ± 19.6	45.2 ± 19.0	<0.001
Hormonal summary	89.0 ± 13.1	90.5 ± 10.6	0.235
Domain-specific HRQOL subscale			
Urinary subscales			
Function	89.3 ± 12.6	92.8 ± 12.2	0.001
Bother	78.3 ± 16.4	85.5 ± 11.4	<0.001
Incontinence	86.9 ± 15.7	92.7 ± 11.0	<0.001
Irritative/obstructive	83.3 ± 13.0	88.0 ± 10.9	<0.001
Bowel subscales			
Function	91.5 ± 10.5	91.9 ± 9.3	0.715
Bother	93.6 ± 10.4	95.3 ± 7.6	0.065
Sexual subscales			
Function	28.0 ± 20.3	37.8 ± 21.1	<0.001
Bother	54.7 ± 31.1	61.8 ± 27.2	0.018
Hormonal subscales			
Function	85.3 ± 15.8	86.9 ± 13.9	0.307
Bother	93.0 ± 9.9	93.5 ± 9.8	0.636

BCI, bladder contractility index; BOOI, bladder outlet obstruction index; BPH, benign prostate hyperplasia; EPIC, Expanded Prostate Cancer Index Composite; HRQOL, health-related quality of life; IIEF-5, International Index of Erectile Function-5; IPSS, International Prostate Symptom Score; PVR, post-voided residual urine volume; QoL, quality of life.

**Table 3:** Characteristics according to age group.

According to Age Group	<65	≥65, <75	≥75	Overall *p* Value	<65 vs. ≥65, <75	<65 vs. ≥75	≥65, <75 vs. ≥75
3-day voiding diary parameters							
N	134	189	44				
Daytime frequency	6.3 ± 1.9	6.7 ± 2.1	6.9 ± 2.1	0.169	0.294	0.428	1.000
Nocturia	0.8 ± 0.6	1.1 ± 0.8	1.7 ± 1.1	<0.001	<0.001	<0.001	<0.001
Urgency	1.1 ± 1.9	1.4 ± 2.4	2.1 ± 3.6	0.052	1.000	0.045	0.175
Urgency urinary incontinence	0.06 ± 0.46	0.03 ± 0.17	0.01 ± 0.05	0.429	0.837	0.846	1.000
Mean bladder capacity	245.7 ± 77.9	219.2 ± 72.4	211.3 ± 76.4	0.003	0.001	0.027	1.000
Nocturnal polyuria index	0.23 ± 0.17	0.28 ± 0.17	0.34 ± 0.14	<0.001	0.014	<0.001	0.102
Urodynamic study							
N	137	190	48				
Qmax	13.6 ± 5.1	12.3 ± 5.7	9.4 ± 4.3	<0.001	0.088	<0.001	0.004
Voided volume	250.7 ± 80.6	240.5 ± 103.8	194.9 ± 89.8	0.002	0.990	0.001	0.009
PVR	29.1 ± 34.6	42.1 ± 43.1	82.4 ± 74.9	<0.001	0.035	<0.001	<0.001
IDC	25.5%	33.2%	41.7%	0.091			
FSV (mL)	201.1 ± 58.6	209.5 ± 58.6	193.3 ± 53.0	0.198	0.652	1.000	0.326
FDV (mL)	298.4 ± 225.2	279.9 ± 69.2	257.8 ± 56.4	0.308	0.936	0.439	1.000
SDV (mL)	359.6 ± 65.6	351.7 ± 68.3	335.3 ± 58.0	0.171	1.000	0.186	0.560
MCC (mL)	371.9 ± 65.4	361.3 ± 71.6	326.9 ± 78.5	0.001	0.371	<0.001	0.007
BOOI	30.1 ± 19.0	35.3 ± 19.0	35.9 ± 19.1	0.041	0.056	0.241	1.000
BCI	100.7 ± 32.0	101.5 ± 68.0	82.0 ± 29.8	0.077	1.000	0.123	0.084
International Prostate Symptom Score (IPSS)							
N	139	196	48				
IPSS total	9.1 ± 6.5	11.1 ± 6.8	13.1 ± 6.3	0.001	0.022	0.001	0.203
IPSS voiding	5.1 ± 4.4	6.0 ± 4.6	6.8 ± 4.3	0.037	0.170	0.061	0.813
IPSS storage	4.0 ± 2.8	5.1 ± 3.1	6.2 ± 3.5	<0.001	0.008	<0.001	0.058
IPSS QoL	2.2 ± 1.5	2.6 ± 1.6	2.9 ± 1.5	0.021	0.114	0.037	0.728
International Index of Erectile Function-5 (IIEF-5)							
N	139	193	48				
Total score	14.2 ± 6.2	11.2 ± 6.0	9.1 ± 5.5	<0.001	<0.001	<0.001	<0.001
Expanded Prostate Cancer Index Composite							
HRQOL domain summary score							
N	139	196	48				
Urinary	86.7 ± 13.4	84.4 ± 13.0	83.2 ± 12.8	0.180	0.370	0.358	1.000
Bowel	92.4 ± 9.3	93.0 ± 7.5	93.0 ± 8.4	0.773	1.000	1.000	1.000
Sexual	46.4 ± 21.2	38.1 ± 18.5	33.4 ± 16.6	<0.001	<0.001	<0.001	<0.001
Hormonal	88.8 ± 13.6	90.3 ± 10.2	89.8 ± 11.9	0.453	0.709	1.000	1.000
Domain-specific HRQOL subscale							
N	139	196	48				
Urinary subscales							
Function	92.7 ± 12.6	90.5 ± 12.0	88.2 ± 13.8	0.072	0.342	0.097	0.766
Bother	84.1 ± 13.3	80.9 ± 15.2	79.7 ± 15.0	0.070	0.132	0.204	1.000
Incontinence	93.1 ± 11.3	88.3 ± 14.4	86.2 ± 16.2	0.001	0.005	0.008	1.000
Irritative/obstructive	86.8 ± 11.7	85.3 ± 12.4	83.9 ± 12.9	0.307	0.784	0.483	1.000
Bowel subscales							
Function	91.3 ± 10.5	92.1 ± 9.0	91.3 ± 11.7	0.716	1.000	1.000	1.000
Bother	94.7 ± 9.2	94.2 ± 8.9	94.8 ± 9.8	0.828	1.000	1.000	1.000
Sexual subscales							
Function	40.8 ± 21.3	30.1 ± 19.5	22.1 ± 20.7	<0.001	<0.001	<0.001	0.045
Bother	60.4 ± 29.1	56.2 ± 29.2	61.1 ± 30.8	0.350	0.610	1.000	0.913
Hormonal subscales							
Function	84.7 ± 16.4	86.4 ± 14.0	89.0 ± 13.6	0.224	0.938	0.277	0.876
Bother	93.3 ± 8.6	93.6 ± 9.4	91.7 ± 14.1	0.490	1.000	1.000	0.700

## Data Availability

The data presented in this study are available on request from the corresponding author. The data are not publicly available due to privacy concerns.

## References

[1] Jung K.W., Won Y.J., Kang M.J., Kong H.J., Im J.S., Seo H.G. (2022). Prediction of Cancer Incidence and Mortality in Korea, 2022. Cancer Res. Treat..

[2] (2022). Statistics Korea [Internet] Daejeon: Statistics Korea. http://kosis.kr.

[3] Urkmez A., Ranasinghe W., Davis J.W. (2020). Surgical techniques to improve continence recovery after robot-assisted radical prostatectomy. Transl. Androl. Urol..

[4] Patel V.R., Coelho R.F., Chauhan S., Orvieto M.A., Palmer K.J., Rocco B., Sivaraman A., Coughlin G. (2010). Continence, potency and oncological outcomes after robotic-assisted radical prostatectomy: Early trifecta results of a high-volume surgeon. BJU Int..

[5] Ficarra V., Novara G., Rosen R.C., Artibani W., Carroll P.R., Costello A., Menon M., Montorsi F., Patel V.R., Stolzenburg J.-U. (2012). Systematic review and meta-analysis of studies reporting urinary continence recovery after robot-assisted radical prostatectomy. Eur. Urol..

[6] Yao H.H.-I., Crump R.T., Charbonneau C., Khan A., Barton C., Brotherhood H., Jiang J., Carlson K.V., Baverstock R.J. (2020). Baseline patient reported outcomes data shows high prevalence of overactive bladder, sexual dysfunction, depression and anxiety in Canadian men with newly diagnosed localized prostate cancer. Transl. Androl. Urol..

[7] Yoo T.K., Lee K.S., Sumarsono B., Kim S.T., Kim H.J., Lee H.C., Kim S.H. (2018). The prevalence of lower urinary tract symptoms in population aged 40 years or over, in South Korea. Investig. Clin. Urol..

[8] Yao H.H., Hoe V., Crump R.T., Sengupta S., O’Connell H.E., Carlson K.V., Baverstock R.J. (2021). Impact of radical prostatectomy on bladder function as demonstrated on urodynamics study-A systematic review. Neurourol. Urodyn..

[9] Hwang Y.C., Cho S.Y., Ku J.H., Jeong S.J., Oh S.J. (2021). Translation and Linguistic Validation of Korean Version of the Expanded Prostate Cancer Index Composite for Clinical Practice for Patients with Prostate Cancer. Int. Neurourol. J..

[10] Emeruwa C.J., Epstein M.R., Michelson K.P., Monaghan T.F., Weiss J.P. (2020). Prevalence of the nocturnal polyuria syndrome in men. Neurourol. Urodyn..

[11] Constantinou C.E., Freiha F.S. (1992). Impact of radical prostatectomy on the characteristics of bladder and urethra. J. Urol..

[12] Kleinhans B., Gerharz E., Melekos M., Weingärtner K., Kälble T., Riedmiller H. (1999). Changes of urodynamic findings after radical retropubic prostatectomy. Eur. Urol..

[13] Aboseif S.R., Konety B., Schmidt R.A., Goldfien S.H., Tanagho E.A., Narayan P.A. (1994). Preoperative urodynamic evaluation: Does it predict the degree of urinary continence after radical retropubic prostatectomy?. Urol. Int..

[14] Rosen R.C., Giuliano F., Carson C.C. (2005). Sexual dysfunction and lower urinary tract symptoms (LUTS) associated with benign prostatic hyperplasia (BPH). Eur. Urol..

[15] Sherer B.A., Levine L.A. (2014). Current management of erectile dysfunction in prostate cancer survivors. Curr. Opin. Urol..

